# Residual depression symptoms following nature-based exercise therapies among U.S. service members

**DOI:** 10.3389/fphys.2026.1788999

**Published:** 2026-07-20

**Authors:** Nicholas P. Otis, Lisa H. Glassman, Alexander C. Kline, Kim T. Kobayashi Elliott, Betty Michalewicz-Kragh, Kristen H. Walter

**Affiliations:** 1 Psychological Health & Readiness, Naval Health Research Center, San Diego, CA, United States; 2 Leidos, Inc., San Diego, CA, United States; 3 Department of Public Health, Naval Medical Center San Diego, San Diego, CA, United States

**Keywords:** adjunctive interventions, blue space, green exercise, military mental health, nature exposure, outdoor physical activity, recreation therapy, treatment outcomes

## Abstract

**Introduction:**

Across interventions for major depressive disorder (MDD), symptoms often persist after treatment. These residual symptoms increase risk for depression relapse and reduce quality of life. Exercise is a common adjunctive intervention for MDD with potential to reduce common residual depression symptoms, yet the frequencies and types of residual symptoms following exercise interventions have seldom been examined and could inform recommendations for maximizing response.

**Methods:**

This study was a secondary analysis of a randomized clinical trial comparing weekly exercise therapy programs (i.e., surf or hike) among 96 active duty service members with MDD. Symptom measures were completed at preprogram, postprogram, and 3-month follow-up. Among the 88 service members who completed a postprogram assessment, analyses examined whether exercise therapy type and loss of diagnosis were related to the odds of reporting each residual depression symptom.

**Results:**

Both interventions significantly improved depression symptoms. Exercise therapy type was not related to the odds of any individual residual symptom (*p*s ≥ .225). Loss of MDD diagnosis reduced the odds of residual depression symptoms (*p*s ≤ .039), except for suicidal ideation at 3-month follow-up (*p* = .088). Sleep difficulties (56%), low energy (38%), and appetite changes (22%) remained common at postprogram among participants who lost their diagnosis. Secondary analyses examined residual posttraumatic stress disorder (PTSD) symptoms in the subgroup with this comorbidity (*n* = 58, 66%). PTSD results showed similar patterns, with sleep disturbance (42%) frequently persisting at postprogram even among those who lost their PTSD diagnosis.

**Conclusion:**

Residual depression symptoms were commonly transdiagnostic and mirrored those reported following evidence-based depression treatments. Additional strategies for reducing residual symptoms following weekly exercise therapy may be warranted for maximum symptom reduction.

## 1 Introduction

Evidence-based treatments for major depressive disorder (MDD), such as cognitive behavioral therapy or selective serotonin reuptake inhibitors, are effective alone or in combination and often produce large reductions in depression symptoms (Cipriani et al., 2018; Cuijpers et al., 2021; Department of Veterans Affairs [VA] et al., 2022). Despite the efficacy of these interventions, over half of individuals who receive evidence-based treatment do not respond (i.e., achieve a 50% reduction in symptoms), and approximately two-thirds do not lose their diagnosis (Cuijpers et al., 2021). Further, symptoms often persist following treatment for depression, even among those who have lost their depression diagnosis (Zimmerman et al., 2007). Definitions of residual symptoms vary across the literature (Pastuszak et al., 2024) but are typically defined as symptoms that remain clinically elevated following treatment (Kovacevic et al., 2022; Larsen et al., 2019). The most common residual symptoms following depression treatment include fatigue, sleep difficulties, and lack of concentration (Conradi et al., 2011; Menza et al., 2003).

Residual symptoms are an important target for depression interventions due to their association with poor long-term outcomes and functioning. The risk of depression relapse is significantly higher in individuals with residual symptoms compared to those without (Nierenberg et al., 2010), and the risk of developing treatment-resistant depression is greater among those with residual symptoms of MDD (Hirschfeld et al., 1997). For example, in a study of treatment-seeking patients with MDD followed naturalistically for over 10 years, those who experienced residual symptoms after treatment relapsed more than three times faster than those without residual symptoms (Judd et al., 1998). Residual symptoms can also cause significant functional impairment, even when patients respond to treatment (Romera et al., 2014; Xiao et al., 2018).

The elevated clinical risk associated with residual symptoms is of particular concern within certain populations, such as active duty service members, who not only face higher rates of MDD relative to the general population (Gaderman et al., 2012), but also unique stressors such as deployments, combat exposure, demanding schedules, and frequent relocations. Such stressors are associated with increased mental health symptom severity and impaired functional status (Fink et al., 2022) and may influence the presence of residual depression symptoms (e.g., sleep difficulties, fatigue, concentration problems), leading to decreased operational readiness and performance. By identifying common residual symptoms among service members with MDD, targeted interventions can be developed to reduce relapse risk, enhance functioning following treatment, and assist with timely return-to-duty.

Most studies of residual depression symptoms have examined traditional treatments such as psychotherapy and pharmacotherapy. However, exercise-based interventions are often effective in treating depression symptoms, are a recommended treatment for depression in the VA/Department of Defense (DoD) MDD Treatment Guidelines (2022), and are increasing in popularity (Wang et al., 2025). Meta-analytic reviews indicate that exercise therapy improves symptoms of MDD (e.g., Morres et al., 2018; Schuch et al., 2016), with moderate between-treatment effect sizes compared to usual care (Kvam et al., 2016). Further, exercise therapies may provide the unique opportunity to improve symptoms of depression that often persist following psychotherapy and pharmacotherapy.

The therapeutic potential of exercise-based interventions is supported by the broad-spectrum neurophysiological adaptations initiated by physical activity, which target pathways distinct from standard pharmacotherapy and psychotherapy. Literature suggests that physical exertion triggers immediate monoaminergic shifts and endorphin release that boost post-session affect (Basso and Suzuki, 2016), while routine exercise over time downregulates hypothalamic-pituitary-adrenal (HPA) axis hyperreactivity and upregulates neurotrophic signaling via brain-derived neurotrophic factor (Yuan, 2026). Through these combined central and autonomic pathways, exercise directly influences neuroinflammation (Yuan, 2026), offering a robust biological rationale for its capacity to improve sleep (Brupbacher et al., 2021a; Khazaie et al., 2023), energy, and fatigue (Niedermeier et al., 2017; Ryan et al., 2010), which are all common residual symptoms following treatments for MDD (McClintock et al., 2011; Menza et al., 2003; Xiao et al., 2018). Given these biological underpinnings, exercise remains an important option for therapeutic intervention.

The way in which exercise therapies are delivered, such as the setting, may also shape its effectiveness. Exercise in natural environments may confer greater psychological benefits for depression than exercise indoors or in urban settings (Barton and Pretty, 2010; Thompson Coon et al., 2011; Wicks et al., 2022). Individual studies and systematic reviews have examined participation in wilderness recreation programs, which include various types of outdoor exercise (e.g., surfing, hiking) and exposure to natural and urban spaces. In these studies, exercise in the natural environment improved symptoms of depression (e.g., Lackey et al., 2021; Rosa et al., 2023; Townsend et al., 2018; Walter et al., 2019b; Walter et al., 2021), including among participants with MDD (Walter et al., 2023b). Reducing residual depression symptoms through exercise—particularly in natural settings—could inform treatment planning, reduce relapse risk, and improve functioning. However, despite these potential benefits, the types and rates of residual symptoms following exercise interventions, including those outdoors and among military populations, remain unknown.

Residual symptoms are also a clinical challenge in common comorbidities with MDD, such as posttraumatic stress disorder (PTSD; Campbell et al., 2007; Green et al., 2006). Service members and veterans with PTSD often report that sleep disturbances, hypervigilance, and concentration problems persist after trauma-focused treatments (Kline et al., 2024, 2025; Kovacevic et al., 2022; Larsen et al., 2019). Similar to MDD, residual symptoms following PTSD treatment are associated with poor functional outcomes and increased relapse risk (Bryant et al., 2016). Exercise interventions, including those delivered in outdoor or natural environments, have also shown promise for improving PTSD symptoms (Greer and Vin-Raviv, 2019; Rosenbaum et al., 2015). Yet, as with MDD, the persistence of residual PTSD symptoms after such interventions, including among service members, has not been examined.

Understanding which symptoms of MDD and PTSD are likely to remain after exercise therapy could clarify whether exercise offers unique transdiagnostic benefits for alleviating common residual symptoms or whether additional symptom-specific interventions are needed to address these symptoms. Critically, no studies to date have examined residual symptoms of either disorder following exercise therapy interventions with active duty service members. This is an important gap given treatment needs for this population, as active duty military are at heightened risk for MDD (Gaderman et al., 2012) and PTSD (Judkins et al., 2020). The primary purpose of this study was to examine the frequency and types of residual depression symptoms following surf or hike therapy among active duty service members with MDD, and to explore whether exercise therapy types differed in their effects on residual symptoms. We also examined the frequencies and odds of residual symptoms between service members who lost MDD diagnosis at postprogram and those who did not. As a secondary aim, we conducted an analysis of residual PTSD symptoms among the subgroup of service members with MDD and comorbid PTSD to determine whether patterns paralleled those observed for MDD alone. Data were derived from a randomized clinical trial (RCT) comparing surf and hike therapies on depression outcomes, which was originally designed to test whether differential effects emerged from a water-based versus land-based activity. Results from the parent study showed that both exercise modalities improved symptoms of depression among service members with MDD (Walter et al., 2023b). Given the exploratory nature of this study and its novelty, no hypotheses were made about the specific residual symptoms or group differences that might emerge.

## 2 Methods

### 2.1 Program

The surf and hike therapy programs were provided as an option within standard care at Naval Medical Center San Diego (NMCSD). These programs were run once per week for 6 weeks. Sessions lasted 3–4 hours and occurred in a cohort-style format of approximately 20 active duty service members per cycle. Psychotherapy techniques (e.g., cognitive restructuring) were not delivered as part of the program; rather, participation in the activity was the therapeutic component (e.g., Hawkins et al., 2016). In the surf therapy program, each service member was paired with a volunteer surf instructor for the duration of the program. All instructors attended an initial clinic orientation and were certified by the Armed Services Young Men’s Christian Association. The surf therapy program was managed and coordinated by a Master’s level exercise physiologist. In the hike therapy program, service members hiked with their cohort. The program manager of the hike therapy program was a Certified Therapeutic Recreation Specialist. Surf and hike therapies occurred in outdoor, public locations in San Diego County.

### 2.2 Participants

Participants in the parent study were 110 active duty service members referred to the Wounded, Ill, and Injured Program at NMCSD between January 2018 and March 2020. After eligibility screening, 96 service members were enrolled in the parent study, with 48 randomized to each therapy condition using blocked randomization. The current study included the 88 service members who completed a postprogram assessment. Inclusion criteria required a Diagnostic and Statistical Manual of Mental Disorders, Fifth Edition (DSM-5; American Psychiatric Association, 2013) diagnosis of MDD as assessed with the Mini International Neuropsychiatric Interview version 7.0 (MINI-7; Sheehan et al., 1998) by a study assessor at preprogram. The sole exclusion criterion was previous participation in the surf or hike therapy programs. Service members received medical clearance to participate in the programs from a medical provider.

### 2.3 Procedure

Service members completed clinical interviews and self-report measures at three assessment points: preprogram (within 2 weeks prior to starting), postprogram (within 2 weeks after program completion), and 3-month follow-up. Assessments were conducted by assessors blinded to treatment condition. Study procedures were approved by the NMCSD Institutional Review Board, and all participants provided written informed consent. Additional details on the parent RCT, including the exercise therapy programs and the CONSORT flowchart, are available elsewhere (Walter et al., 2019a; Walter et al., 2023b).

### 2.4 Measures

The primary outcome, depression symptom severity, was assessed using the 9-item Patient Health Questionnaire (PHQ-9; Kroenke et al., 2001). The PHQ-9 is a commonly used, validated self-report measure reflecting DSM-5 criteria for MDD, including symptoms such as anhedonia, depressed mood, sleep, energy, and appetite. Each item is scored from 0–3 (total range 0–27), where higher scores indicate greater depression symptom severity. Internal consistency in this sample ranged from good to excellent (α = 0.77–0.89). MDD diagnosis at each timepoint was determined using the MINI-7.

To examine the secondary aim, PTSD symptom severity was assessed using the PTSD Checklist for DSM-5 (PCL-5; Weathers et al., 2013b) with the extended Life Events Checklist (Weathers et al., 2013a) used to identify participants’ index trauma. The PCL-5 is a widely used, 20-item self-report measure with strong psychometrics (Bovin et al., 2016) that directly aligns with DSM-5 criteria for PTSD. Each item is scored from 0–4 (total range 0–80), with higher scores suggesting greater PTSD symptom severity. Internal consistency was excellent in this sample (α = 0.87–0.97). PTSD diagnosis at each timepoint was determined using the MINI-7.

At preprogram, participants also self-reported demographics, physical activity (via the 7-item International Physical Activity Questionnaire–Short Form; IPAQ-SF; Craig et al., 2003), and engagement in concurrent depression treatment.

### 2.5 Analysis

A residual symptom was coded as present if the participant endorsed a clinically significant symptom at both pre- and postprogram assessments, following the methodology used in prior publications studying residual symptoms (e.g., Kline et al., 2024, 2025; Kovacevic et al., 2022; Larsen et al., 2019). For 3-month follow-up analyses, symptoms needed to be present at all three assessment timepoints (Kovacevic et al., 2022). This coding scheme ensured the identification of persistent—not emergent—symptoms. The threshold for a clinically significant symptom was specific to the measure used and consistent with published methodology (e.g., Kovacevic et al., 2022). On the PHQ-9, a clinically significant depression symptom was defined as (a) items 1–8 rated a 2 (*more than half the days*) or above or (b) item 9 (i.e., suicidal ideation) rated 1 (*several days*) or above. On the PCL-5, a clinically significant PTSD symptom was defined as an item rated 2 (*moderately*) or above. Diagnostic status was defined as no longer meeting MDD or PTSD diagnostic criteria based on the MINI-7 at postprogram.

For each symptom, binary logistic regressions tested effects of (a) randomized treatment group and (b) diagnostic status at postprogram and 3-month follow-up. If logistic regressions were not possible due to low cell counts (i.e., <5), Fisher’s Exact Tests were used, in line with previous research (Tripp et al., 2020b). To preserve statistical robustness and avoid the risk of floor effects, 3-month follow-up analyses for residual PTSD symptoms were omitted, as most of these items yielded insufficient cell counts. Because multiple tests were run, Hochberg’s step-up procedure (Hochberg, 1988) was applied to reduce Type I error, mirroring prior work on residual symptoms (e.g., Kline et al., 2024, 2025; Kovacevic et al., 2022). Rates of missing data were 8.3% (*n* = 8) at postprogram and 21.9% (*n* = 21) at 3-month follow-up. Participants with complete data did not significantly differ compared to those with missing data on preprogram depression severity scores or any demographic variables. To handle missing data and examine consistency in our results, we also conducted multiple imputation and ran identical analyses with these data. Results using multiple imputation did not differ from those of the original dataset; thus, results from analyses using available data are reported here. All analyses were completed using SPSS Statistics version 29 (IBM, Armonk, NY).

## 3 Results

### 3.1 Residual depression symptoms

**Table 1** displays sample characteristics by exercise therapy type and loss of MDD diagnosis. Service members were relatively young (*M* = 28.1 years, *SD* = 5.6) and physically active (72% at least moderately active per IPAQ scoring guidelines [Herrmann et al., 2024]). Approximately 91% reported receiving concurrent psychotherapy or pharmacotherapy for depression. Overall, the sample self-reported moderately severe depression symptom severity based on PHQ-9 scores (*M* = 17.1, *SD* = 4.9). Service members randomized to surf therapy had significantly lower preprogram PHQ-9 scores than those randomized to hike therapy (*MD* = −2.2, *p* = .025), although this difference was not clinically significant (Turkoz et al., 2021).

**Table 1:** Preprogram sample characteristics.

Characteristic	Total sample	Surf therapy	Hike therapy	Lost MDD diagnosis^a^	Retained MDD diagnosis^a^
	*N* = 88	*n* = 47	*n* = 41	*n* = 45	*n* = 43
Age, years, *M* (*SD*)	28.4 (5.8)	29.3 (6.2)	27.4 (5.0)	29.3 (5.9)	27.5 (5.6)
Sex, *n* (%)					
Women	45 (51.1)	22 (46.8)	23 (56.1)	24 (53.3)	21 (48.8)
Men	43 (48.9)	25 (53.2)	18 (43.9)	21 (46.7)	22 (51.2)
Race/ethnicity, *n* (%)					
Asian or Asian-American	2 (2.3)	2 (4.3)	0 (0.0)	2 (4.4)	1 (2.3)
Black or African American	13 (14.8)	6 (12.8)	7 (17.1)	8 (17.8)	5 (11.6)
Hispanic, Latino, or Spanish origin	18 (20.5)	8 (17.0)	10 (24.4)	8 (17.8)	10 (23.3)
Multiracial	18 (20.5)	10 (21.3)	8 (19.5)	9 (20.0)	9 (20.9)
Native American or Alaska Native	1 (1.1)	0 (0.0)	1 (2.4)		
White	36 (40.9)	21 (44.7)	15 (36.6)	18 (40.0)	18 (23.3)
Rank, *n* (%)					
E1–E4	30 (34.1)	13 (27.7)	17 (41.5)	12 (26.7)	18 (41.9)
E5–E9	53 (60.2)	32 (68.0)	21 (51.2)	30 (66.6)	23 (53.5)
Officer	5 (5.2)	2 (4.3)	3 (7.3)	3 (6.6)	2 (4.7)
Concurrent depression treatment, *n* (%)	80 (90.9)	43 (91.5)	37 (90.2)	41 (91.1)	39 (90.7)
Pharmacotherapy	59 (67.0)	32 (68.1)	27 (65.9)	29 (64.4)	30 (69.8)
Psychotherapy	79 (89.8)	42 (89.4)	37 (90.2)	41 (91.1)	38 (88.4)
Activity level, *n* (%)^b^					
Low/inactive (<600 MET mins/wk)	10 (11.4)	8 (17.0)	2 (4.9)	4 (8.9)	6 (14.0)
Moderately active (600–2999 MET mins/wk)	31 (35.2)	18 (38.3)	13 (31.7)	16 (35.6)	15 (34.9)
Highly active (≥3000 MET mins/wk)	31 (35.2)	13 (27.7)	18 (43.9)	15 (33.3)	16 (37.2)
Sessions attended, *M* (*SD*)^c^^,^ ^d^	3.7 (1.8)	3.5 (1.7)	3.9 (1.9)	3.8 (1.6)	3.5 (1.9)
Completion of assigned program, *n* (%)^d^^,^ ^e^	63 (78.8)	36 (83.7)	27 (73.0)	34 (82.9)	29 (74.4)
Depression severity (PHQ-9), *M* (*SD*)	17.0 (4.8)	15.9 (4.8)^*^	18.3 (4.6)^*^	15.6 (5.1)^**^	18.5 (4.1)^**^
PTSD severity (PCL-5), *M (SD)^f^*	50.3 (13.3)	46.5 (12.7)	52.9 (13.3)	46.5 (14.4)	52.5 (11.8)

E, enlisted rank; MDD, major depressive disorder; MET mins, metabolic equivalent minutes; PHQ-9 = 9-item Patient Health Questionnaire. Counts vary based on missing data; percentages are based on full samples for that column.

^a^ At postprogram assessment.

^b^ Physical activity level data were calculated (see IPAQ Research Committee, 2005) from the self-report version of the International Physical Activity Questionnaire–Short Form.

^c^ For fidelity, only sessions in which the assigned modality was conducted are included. Occasionally, due to adverse weather, sessions consisted of alternative activities (e.g., visit to National Surf Museum, strength and conditioning class in Hiking Therapy).

^d^ Because programs were halted due to the sudden onset of COVID-19, participants (*n* = 8) in the affected cohort were not counted in completion or attendance statistics.

^e^ Program completion was defined by NMCSD as missing no more than two sessions of the assigned modality.

^f^ Among those with a PTSD diagnosis only.

*p* <.05; ^**^p <.01.

**Table 2** displays frequencies of residual depression symptoms for the overall sample. At postprogram, the most common residual depression symptoms in the full sample included sleep difficulties (66%), low energy (60%), and appetite changes (40%). Most (82%) participants endorsed at least one residual symptom at postprogram. These same residual symptoms remained the most prevalent at 3-month follow-up. The largest symptom decreases from preprogram to 3-month follow-up were observed for depressed mood (-58%), loss of interest (-55%), and low self-worth (-50%).

**Table 2:** Frequencies and conditional probabilities of residual depression symptoms (PHQ-9) overall and between randomized exercise groups.

Depression symptom	Total sample		Surf therapy		Hike therapy			
	%	*n*	%	*n*	%	*n*	OR [95% CI]	*p^a^*
Postprogram (*n* = 88)								
Lack of interest	31.8	28	23.4	11	41.5	17	1.47 [0.52, 4.17]	.356
Down	37.9	33	30.4	14	46.3	19	1.28 [0.48, 3.44]	.356
Sleep change	65.9	58	61.7	29	70.7	29	0.87 [0.30, 2.48]	.429
Energy change	60.2	53	59.6	28	61.0	25	0.61 [0.22, 1.66]	.356
Appetite change	39.8	35	36.2	17	43.9	18	0.88 [0.34, 2.31]	.919
Low self-esteem	36.4	32	25.5	12	48.8	20	1.79 [0.61, 5.25]	.356
Concentration	33.0	29	36.2	17	29.3	12	0.41 [0.15, 1.16]	.356
Psychomotor	17.0	15	14.9	7	19.5	8	1.00 [0.31, 3.24]	.969
Suicidal ideation	17.0	15	12.8	6	22.0	9	1.25 [0.37, 4.21]	.919
3-month follow-up (*n* = 74)								
Lack of interest	20.3	15	11.6	5	32.3	10	2.47 [0.70, 8.75]	.225
Down	19.2	14	14.3	6	25.8	8	1.33 [0.38, 4.74]	.771
Sleep change	54.1	40	46.5	20	64.5	20	1.28 [0.44, 3.71]	.376
Energy change	41.9	31	37.2	16	48.4	15	0.93 [0.32, 2.68]	.776
Appetite change	27.0	20	25.6	11	29.0	9	0.60 [0.18, 1.99]	.771
Low self-esteem	17.6	13	9.3	4	18.8	9	–^b^	.225
Concentration	23.0	17	25.6	11	19.4	6	0.26 [0.07, 1.04]	.363
Psychomotor	12.2	9	9.3	4	16.1	5	–^b^	.717
Suicidal ideation	8.1	6	4.7	2	12.9	4	–^b^	.414

CI, confidence interval; OR, odds ratio; PHQ-9, 9-item Patient Health Questionnaire. Logistic regressions for the PHQ-9 controlled for preprogram scores, as these were significantly different between randomized exercise groups. Because Surf Therapy is the reference group, an OR <1 indicates lower odds for Hike Therapy compared with Surf Therapy, and an OR >1 indicates higher odds for Hike Therapy.

^a^ Hochberg-adjusted *p* values.

^b^ Fisher’s Exact Test.

**Table 2** also displays frequencies and odds of endorsing a residual depression symptom in relation to exercise therapy group. Controlling for preprogram scores, exercise type did not significantly influence the odds of any residual symptom at either timepoint (Hochberg-adjusted *p*s = .225–.969). Surf and hike therapies also did not significantly differ on the overall number of residual symptoms among participants at either timepoint (*p*s = .106–.321).

Roughly half (51%) of the sample (*n* = 45) lost their MDD diagnosis at postprogram. **Table 3** shows frequencies and odds of residual depression symptoms by diagnostic status. Loss of MDD diagnosis was associated with lower odds of all residual symptoms at both timepoints (adjusted *p*s <.001–.039), except for suicidal ideation at follow-up (*p* = .088), likely reflecting a floor effect due to low endorsement (3%). Among those who lost their MDD diagnosis at postprogram, the most common residual symptoms were sleep difficulty (56%), low energy (38%), and appetite changes (22%), consistent with the broader sample. On average, service members who lost their MDD diagnosis endorsed 1.9 (SD = 2.0) residual depression symptoms at postprogram, compared to 4.9 (SD = 2.6) among those who continued to meet diagnostic MDD criteria.

**Table 3:** Frequencies and conditional probabilities of residual symptoms (PHQ-9) between MDD diagnostic status groups.

Depression symptom	Lost MDD diagnosis		Retained MDD diagnosis			
	%	*n*	%	*n*	OR [95% CI]	*p* ^a^
Postprogram (*n* = 88)						
Lack of interest	8.9	4	55.8	24	–^b^	<.001
Down	15.6	7	61.9	26	8.82 [3.19, 24.43]	<.001
Sleep change	55.6	25	76.7	33	2.64 [1.05, 6.62]	.039
Energy change	37.8	17	83.7	36	8.47 [3.09, 23.24]	<.001
Appetite change	22.2	10	58.1	25	4.86 [1.92, 12.30]	<.001
Low self-esteem	20.0	9	51.2	22	3.67 [1.46, 9.23]	.009
Concentration	20.0	9	46.5	20	3.48 [1.35, 8.95]	.013
Psychomotor	4.4	2	30.2	13	–^b^	.002
Suicidal ideation	6.7	3	27.9	12	–^b^	.011
3-month follow-up (*n* = 74)						
Lack of interest	0.0	0	44.1	15	–^b^	<.001
Down	2.5	1	39.4	13	–^b^	<.001
Sleep change	40.0	16	70.6	24	3.60 [1.36, 9.51]	.011
Energy change	22.5	9	64.7	22	6.32 [2.27, 17.56]	<.001
Appetite change	12.5	5	44.1	15	5.53 [1.74, 17.56]	.006
Low self-esteem	2.5	1	35.3	12	–^b^	<.001
Concentration	7.5	3	41.2	14	–^b^	<.001
Psychomotor	2.5	1	23.5	8	–^b^	.011
Suicidal ideation	2.5	1	14.7	5	–^b^	.088

CI, confidence interval; MDD, major depressive disorder; OR = odds ratio; PHQ-9, 9-item Patient Health Questionnaire. Because remitted (i.e., loss of diagnosis) is the reference group, an OR <1 indicates lower odds for the non-remitted (i.e., retained) group compared with the remitted group, and an OR >1 indicates higher odds for the non-remitted (i.e., retained) group.

^a^ Hochberg-adjusted *p* values.

^b^ Fisher’s Exact Test.

### 3.2 Subgroup analysis: residual PTSD symptoms

Of the 88 service members with MDD, most (66%; *n* = 58) also met diagnostic criteria for PTSD, prompting a secondary analysis of residual PTSD symptoms. The most common index trauma among those with comorbid PTSD was sexual assault (55%), and the mean PCL-5 score of this subgroup was 50.3 (SD = 13.3), indicating moderately severe PTSD.

Among service members with comorbid PTSD, the most common residual PTSD symptoms reported at the postprogram assessment were sleep difficulties (52%), cued distress (40%), and avoidance of trauma reminders (35%). At postprogram, participants reported an average of 4.6 (*SD* = 4.9) residual symptoms, and 72% of participants reported at least one residual PTSD symptom. Surf and hike therapies did not differ on the overall number of residual PTSD symptoms at program end (*p*s = .728). As shown in **Table 4**, exercise type did not influence the odds of having any specific residual PTSD symptom (adjusted *p*s = .906–.999).

**Table 4:** Frequencies and conditional probabilities of residual PTSD symptoms (PCL-5) overall and between exercise conditions.

PTSD symptom	Total sample		Surf therapy		Hike therapy			
	%	*n*	%	*n*	%	*n*	OR [95% CI]	*p^a^*
Postprogram (*n* = 54)								
B1. Intrusive memories	0.0	0	0.0	0	0.0	0	--	--
B2. Nightmares	18.5	10	16.7	4	20.0	6	--^b^	.999
B3. Flashbacks	9.3	5	4.2	1	13.3	4	--^b^	.906
B4. Cued distress	39.6	21	37.5	9	41.4	12	1.18 [0.39, 3.57]	.999
B5. Cued physical reactions	31.5	17	33.3	8	30.0	9	0.86 [0.27, 2.72]	.999
C1. Avoid thoughts	34.0	18	33.3	8	34.5	10	1.05 [0.34, 3.30]	.999
C2. Avoid reminders	35.2	19	29.2	7	40.0	12	1.62 [0.52, 5.08]	.906
D1. Trauma amnesia	13.2	7	12.5	3	13.8	4	--^b^	.999
D2. Negative beliefs	22.2	12	12.5	3	30.0	9	--^b^	.906
D3. Blame	22.2	12	20.8	5	23.3	7	1.16 [0.32, 4.24]	.999
D4. Negative feelings	23.1	12	26.1	6	20.7	6	0.74 [0.20, 2.70]	.999
D5. Loss of interest	31.5	17	20.8	5	40.0	12	2.53 [0.74, 8.64]	.906
D6. Detachment	30.2	16	26.1	6	33.3	10	1.42 [0.43, 4.71]	.999
D7. Numbing	11.1	6	12.5	3	10.0	3	--^b^	.999
E1. Irritability or aggression	22.6	12	13.0	3	30.0	9	--^b^	.906
E2. Reckless behavior	5.6	3	0.0	0	10.0	3	--^b^	.906
E3. Hypervigilance	33.3	18	25.0	6	40.0	12	2.00 [0.62, 6.49]	.906
E4. Startle	31.5	17	29.2	7	33.3	10	1.21 [0.38, 3.88]	.999
E5. Concentration difficulties	31.5	17	37.5	9	26.7	8	0.61 [0.19, 1.93]	.906
E6. Sleep difficulties	51.9	28	45.8	11	56.7	17	1.55 [0.53, 4.55]	.906

PTSD, posttraumatic stress disorder. To preserve statistical robustness and avoid the risk of severe floor effects, 3-month follow-up analyses for residual PTSD symptoms were omitted, as the vast majority of these items yielded insufficient cell counts.

^a^ Hochberg-adjusted *p* values.

^b^ Fisher’s exact test.

Approximately half (54%; *n* = 31) of the 58 service members with comorbid PTSD lost their diagnosis by postprogram. **Table 5** displays residual PTSD symptom frequencies and odds in accordance with diagnostic status. Service members who lost their PTSD diagnosis mainly continued to experience sleep difficulties (42%). Loss of PTSD diagnosis was associated with reduced odds of most residual symptoms at postprogram. Symptoms that did not yield significantly different odds due to diagnostic status were those with very low cell counts in each group, again likely reflecting floor effects.

**Table 5:** Frequencies and conditional probabilities of residual PTSD symptoms (PCL-5) between PTSD diagnostic status groups.

PTSD symptom	Lost PTSD diagnosis		Retained PTSD diagnosis			
	%	*n*	%	*n*	OR [95% CI]	*p^a^*
Postprogram (*n* = 53)						
B1. Intrusive memories	0.0	0	0.0	0	--	--
B2. Nightmares	3.2	1	40.9	9	--^b^	<.001
B3. Flashbacks	3.2	1	18.2	4	--^b^	.147
B4. Cued distress	16.1	5	76.2	16	16.64 [4.16, 66.62]	<.001
B5. Cued physical reactions	9.7	3	63.6	14	--^b^	<.001
C1. Avoid thoughts	16.1	5	61.9	13	8.45 [2.30, 31.03]	<.001
C2. Avoid reminders	12.9	4	68.2	15	--^b^	<.001
D1. Trauma amnesia	3.2	1	28.6	6	--^b^	.019
D2. Negative beliefs	6.5	2	45.5	10	--^b^	.003
D3. Blame	6.5	2	45.5	10	--^b^	.003
D4. Negative feelings	9.7	3	45.0	9	--^b^	.010
D5. Loss of interest	12.9	4	59.1	13	--^b^	<.001
D6. Detachment	16.1	5	52.4	11	5.72 [1.58, 20.66]	.070
D7. Numbing	3.2	1	22.7	5	--^b^	.075
E1. Irritability or aggression	10.0	3	40.9	9	--^b^	.023
E2. Reckless behavior	0.0	0	13.6	3	--^b^	.074
E3. Hypervigilance	16.1	5	59.1	13	7.51 [2.09, 27.01]	<.001
E4. Startle	12.9	4	59.1	13	--^b^	<.001
E5. Concentration difficulties	12.9	4	59.1	13	--^b^	<.001
E6. Sleep difficulties	41.9	13	68.2	15	2.97 [0.94, 9.34]	.074

PTSD, posttraumatic stress disorder. To preserve statistical robustness and avoid the risk of severe floor effects, 3-month follow-up analyses for residual PTSD symptoms were omitted, as most of these items yielded insufficient cell counts.

^a^ Hochberg-adjusted *p* values.

^b^ Fisher’s exact test.

## 4 Discussion

Even among patients who respond well to treatment for depression, a sizeable portion of individuals with MDD experience residual symptoms, which can impair functioning and increase relapse risk. The frequencies and types of these depression symptoms after exercise-based interventions, however, are largely unknown, especially in the active duty military population. Study results showed that most service member participants (82%) reported at least one residual depression symptom after surf or hike therapy, with sleep difficulties (66%), low energy (60%), and appetite changes (40%) the most frequently endorsed. The likelihood of individual residual depression symptoms did not significantly differ between surf and hike therapy. Although loss of MDD diagnosis reduced the odds of most residual symptoms, several non-specific symptoms (i.e., sleep difficulties, low energy, appetite changes) remained elevated among this group. These findings suggest that these somatic symptoms may not be adequately addressed with once-weekly surf and hike therapies, and that further intervention may be necessary.

Residual depression symptoms following exercise therapy were similar to those following psychotherapy and pharmacotherapy for depression (Menza et al., 2003) and included difficulties with sleep, energy, and appetite. Although presentations of depression are highly heterogenous—with over 200 symptom combinations that can result in a diagnosis (Zimmerman et al., 2015)—residual symptoms following evidence-based interventions may be consistent across varying populations and treatment types. In line with this, studies of MDD interventions consistently report that sleep problems (Iovieno et al., 2011; Menza et al., 2003; Nierenberg et al., 1999), low energy (Hybels et al., 2005; Menza et al., 2003), and appetite changes (Nierenberg et al., 2010; Xiao et al., 2018) are common residual symptoms. Despite implementing a different type of intervention, results from the current study replicated prior findings. Furthermore, the frequency of residual symptoms among service members who lost their MDD diagnosis in this study were comparable to those found in the literature following psychotherapy and pharmacotherapy. For example, sleep difficulties were still present in 56% of service members in this study who lost their MDD diagnosis, aligning with the 30–67% range observed in prior research (Iovieno et al., 2011; Nierenberg et al., 1999; Xiao et al., 2018). Likewise, low energy was reported among 38% of service members who lost their diagnosis, similar to the 29–42% observed following evidence-based depression treatment (Hybels et al., 2005; Iovieno et al., 2011; Nierenberg et al., 1999; Xiao et al., 2018). Appetite changes (22%) in the current study were slightly lower than the 29–36% reported in other literature (Nierenberg et al., 2010; Xiao et al., 2018). While posttreatment residual symptom rates vary widely across the literature, ranging from as low as 33% up to 98%, the prevalence of participants retaining at least one symptom in this study (87%) aligns closely with the higher estimates documented in several clinical trials (Iovieno et al., 2011; Kovacevic et al., 2022; Nierenberg et al., 2010; Xiao et al., 2018).

Several factors may contribute to the persistence of sleep, energy, and appetite difficulties in this study. Importantly, while these symptoms are included in the DSM-5 MDD criteria, they overlap with many other conditions. Persistent symptoms after treatment may reflect somatic, environmental, or pharmacological factors rather than residual depression alone. For instance, these symptoms may be maintained by other psychological disorders such as anxiety or substance use disorders, or physiological conditions such as thyroid disorders, sleep apnea, or anemia. Further, sleep, energy, and appetite difficulties are common side effects of antidepressants (Kelly et al., 2008); two-thirds of the sample endorsed concurrent pharmacotherapy for depression at preprogram. Ongoing operational or psychosocial stressors unique to the military environment may also drive symptom persistence. For example, approximately 27–54% of military personnel experience symptoms of insomnia (Byrne et al., 2021), possibly due to demanding schedules, operational stress, and poor sleep environments.

Beyond these environmental factors, the structural “dose” of the intervention itself may play a critical role (Gerber et al., 2016). Although regular exercise is known to improve sleep quality, increase energy levels, and regulate appetite (Brupbacher et al., 2021a; Gourgouvelis et al., 2018; Niedermeier et al., 2017; Ryan et al., 2010), the once-weekly, three-hour format used in this trial may not have provided sufficient dose to sustain improvements beyond short-term gains, as acute exercise effects on these symptoms are transient (Brupbacher et al., 2021b; Loy et al., 2013; Thackray and Stensel, 2023; Walter et al., 2019b; Wender et al., 2022). This limitation may be particularly relevant in demanding modalities like surf therapy, which can induce high levels of physical exertion (Otis et al., 2026). In an active duty military sample—where baseline operational stress and sleep deprivation are already high—this intense physical effort could temporarily increase fatigue during the immediate 24-to-72-hour recovery window (Skorski et al., 2019). Without a higher weekly frequency to stimulate long-term physical conditioning, a once-weekly dose may be insufficient for physiological stabilization. Consequently, certain somatic symptoms may lag behind rapid depressive or affective symptom improvements as systemic neuroendocrine and HPA-axis stabilization require a more frequent training stimulus to fully resolve chronic fatigue and sleep issues (Silverman and Deuster, 2014). Due to these biological mechanisms, increased exercise session frequency may be required to move participants beyond acute recovery into long-term physiological adaptation.

Type of exercise therapy, another factor that might influence residual symptoms, was not significantly related to the odds of residual depression symptoms in this study. This mirrors the parent trial’s main findings that both interventions produced similar reductions in overall depression symptom severity (Walter et al., 2019b). Our finding of similar residual symptom profiles for surfing and hiking addresses a key question regarding how nature-based interventions work. Because the parent trial was designed to isolate the unique impacts of blue space exposure (surf therapy) by utilizing an active, green space control (hike therapy), these null findings suggest that the specific environmental context may not differentially influence residual symptoms. Instead, shared characteristics including outdoor environments, physical activity, social settings, and effects such as attention restoration, may drive the primary therapeutic benefits (Bratman et al., 2019; Kaplan, 1995). However, because individual psychological mediators like subjective environmental perception or explicit nature-connectedness were not psychometrically tracked, untangling how these shared restorative environments interact with physical activity to influence the persistence or remission of specific residual symptoms remains a critical direction for future research.

Clinically, the lack of differential effects suggests that participants may benefit equally from a variety of outdoor exercise modalities, making patient preference and accessibility critical considerations in treatment selection. Hiking, for instance, may be more geographically and financially accessible than surfing, while still offering comparable benefits. This shared decision-making approach is a key recommendation in the VA/DoD Treatment Guidelines for MDD (2022). Such flexibility supports a patient-centered approach that tailors adjunctive exercise to individual needs and contexts and may help sustain engagement over time (Busch et al., 2016).

In addition to residual depression symptoms, the current study explored PTSD residual symptoms given the high comorbidity with MDD, including in this sample. In the PTSD subgroup analysis, participants with comorbid PTSD showed similar patterns to those with MDD alone. Specifically, exercise type was not significantly related to the odds of any PTSD residual symptom, and although loss of PTSD diagnosis was linked to reduced odds of most residual symptoms, sleep difficulties remained prevalent. This is consistent with prior research in active duty and veteran samples, which found that sleep difficulties frequently persist following evidence-based PTSD treatment (e.g., Miles et al., 2022; Schnurr and Lunney, 2019), including among those with both PTSD and MDD (Kline et al., 2025). PTSD is highly heterogeneous (Galatzer-Levy and Bryant, 2013), yet this pattern has now been observed across different treatments, samples, and settings, even among those who report meaningful symptom reduction. The shared persistence of sleep difficulties in both MDD and PTSD highlights that this is a transdiagnostic residual symptom and may be maintained by factors beyond the pathology of either disorder (e.g., insomnia; inconsistent military sleep schedules). Consequently, effective clinical management may require a targeted, integrated approach. Clinicians can consider referring service members to specialized providers to assess sleep difficulties. Furthermore, to augment the benefits of exercise therapy, treatment plans could incorporate concurrent or sequenced symptom-specific behavioral interventions like cognitive-behavioral therapy for insomnia (CBT-I) or tailored behavioral management strategies (e.g., structured activity scheduling, energy pacing, and sleep hygiene conditioning) to help service members manage post-exercise fatigue and sleep difficulties. A crucial endeavor for future research is to evaluate these integrated treatment strategies that specifically address the most impairing residual symptoms reported by service members.

Study results should be interpreted with consideration of several limitations. The sample consisted of active duty service members who were relatively young and physically active at the start of the exercise therapy programs. This may heighten the risk of selection bias, and results may not generalize to other populations such as nonmilitary, older, or other populations with depression, all of whom tend to be less physically active. Additionally, outcomes for the current study relied on self-report measures, rather than clinician-rated assessments. Objective sleep data were also not collected. However, both the PHQ-9 and PCL-5 demonstrate strong psychometric properties and these measures are commonly used with service members and in other studies of residual symptoms (e.g., Kline et al., 2024; 2025; Kovacevic et al., 2022). Most participants (91%) reported use of concurrent treatments such as psychotherapy or pharmacotherapy, so exercise therapy was largely delivered as an adjunctive intervention. Prior work from the parent study has shown that these concurrent treatments yielded non-statistically significant relationships with outcomes over time (Walter et al., 2023a, Walter et al., 2023b); therefore, to preserve model parsimony and reduce risk of overfitting, these concurrent treatment variables were excluded from the primary analyses. Furthermore, following methodological precedent in the residual symptom literature, baseline covariates are typically omitted, which facilitates comparisons across clinical trials (e.g., Kline et al., 2024, 2025; Kovacevic et al., 2022). To further ensure this modeling decision did not affect our conclusions, we conducted a sensitivity analyses restricting the sample to participants receiving concurrent depression treatment. This analysis yielded a substantively similar pattern of results, corroborating that concurrent care did not confound study findings. Next, self-reported physical activity data were only collected at preprogram assessment due to concerns regarding whether the IPAQ-SF was suitable to assess individual-level change in small samples (Bauman et al., 2009; Lee et al., 2011). The lack of self-reported physical activity at follow-up time points precludes the ability to examine ongoing engagement and whether other leisure-time physical activity influenced residual symptom outcomes over time. Lastly, though this sample was comparable in size to other research on residual symptoms in clinical trials (e.g., Klein et al., 2025; Kline et al., 2024; Tripp et al., 2020a), the smaller sample size limited statistical power and should be taken into consideration. Several items on the PHQ-9 and PCL-5, such as suicidal ideation, were endorsed at low frequencies that may reflect floor effects. Despite using statistical methods to account for this (e.g., Fisher’s Exact Tests, Hochberg’s step-up procedure), results should be interpreted cautiously.

This study also offers numerous strengths that contribute to the larger literature on exercise and treatment for depression. To our knowledge, this study is the first to examine residual depression and PTSD symptoms following exercise therapy programs. The current study also extends research on residual symptoms to an active duty military population, which have seldom been studied in this literature (see Kline et al., 2024, 2025 for exceptions). Data were derived from an RCT (Walter et al., 2023b) that was both rigorous and pragmatic, thereby increasing confidence in the findings while enhancing external validity. The interventions were delivered as part of standard care at a military treatment facility without altering the hospital’s standard care procedures. Service members completed assessments with strong psychometric properties widely used in military settings, residual symptom research, and that map onto DSM-5 criteria. Multiple imputation was used to account for missing data, and results from both analyses were comparable, increasing confidence in findings. Finally, the methodology of the current study, such as definitions of residual symptoms and statistical analyses, mirrored published studies on residual symptoms to facilitate comparisons across the literature.

## 5 Conclusions

Residual depression symptoms increase the chances of relapse, poor functioning, and physical inactivity for individuals with MDD. Despite a growing body of literature recommending exercise as treatment for depression (Wang et al., 2025), to date, research has yet to adequately examine residual symptoms following these interventions. Collectively, findings suggest that while once-weekly surf and hike therapies produce clinically and statistically significant improvements in overall depression symptom severity, they may not fully resolve common residual symptoms such as sleep, energy, and appetite difficulties. These symptoms correspond to the most common residual symptoms endorsed following widely used, traditional psychotherapies and pharmacotherapies, suggesting that these symptoms may be particularly recalcitrant, maintained by other mental or physical health concerns, or a result of inadequate exercise dosing. Sleep difficulties also emerged as a shared residual PTSD symptom among those with comorbid PTSD, perhaps suggesting that additional sleep-focused interventions may be warranted following treatment if these symptoms persist. Exercise therapy type did not significantly affect residual symptom likelihood, indicating that tailoring modality to patient preference and accessibility may support engagement without compromising benefit. Assessing symptoms at posttreatment is critical to inform tailored, patient-centered strategies and continuity of care following treatment.

## Data Availability

The dataset generated and/or analyzed during the current study are not publicly available due to personally identifiable information regulations, but they may be made available by the corresponding author on reasonable request and approval by the Naval Medical Center San Diego Institutional Review Board/Privacy Office.

## Author disclaimer

KHW, KTKE, and BMK are employees of the U.S. Government. This work was prepared as part of their official duties. Title 17, U.S.C. §105 provides that copyright protection under this title is not available for any work of the U.S. Government. Title 17, U.S.C. §101 defines a U.S. Government work as work prepared by a military service member or employee of the U.S. Government as part of that person’s official duties. Report No. 25–95 was supported by the U.S. Navy Bureau of Medicine and Surgery under work unit no. N1600. The views expressed in this article are those of the authors and do not necessarily reflect the official policy or position of the Department of the Navy, Department of Defense, nor the U.S. Government. The study protocol was approved by the Naval Medical Center San Diego Institutional Review Board in compliance with all applicable federal regulations governing the protection of human subjects. Research data were derived from approved Naval Medical Center San Diego Institutional Review Board protocol number NMCSD.2017.0007.
